# Natural gas odorants: A scoping review of health effects

**DOI:** 10.1007/s40572-023-00403-w

**Published:** 2023-07-25

**Authors:** Drew R. Michanowicz, Olivia M. Leventhal, Jeremy K. Domen, Samuel R. Williams, Eric D. Lebel, Lee Ann L. Hill, Jonathan J. Buonocore, Curtis L. Nordgaard, Aaron S. Bernstein, Seth B.C. Shonkoff

**Affiliations:** 1 Center for Climate, Health and the Global Environment, Harvard T.H. Chan School of Public Health, Boston, MA 02215 United States; 2 PSE Healthy Energy, Oakland, CA 94612 United States; 3 School of Medicine, University of California, San Francisco, CA 94143 United States; 4 Earth & Environmental Sciences Area, Lawrence Berkeley National Lab, Berkeley, CA 94702 United States; 5 Boston University School of Public Health, Boston, MA USA; 6 Division of General Medicine Pediatrics, Boston Children’s Hospital, Boston, MA 02115 United States; 7 Department of Environmental Science, Policy and Management, University of California, Berkeley, Berkeley, CA 94702 United States; 8 Energy Technologies Area, Lawrence Berkeley National Lab, Berkeley, CA 94702 United States

**Keywords:** downstream natural gas, odorants, mercaptans, health effects, community exposure, organosulfur compounds

## Abstract

**Purpose of Review:**

Organosulfur compounds are intentionally added to natural gas as malodorants with the intent of short-term nasal inhalation to aid in leak detection. Regulatory exposure limits have not been established for all commonly used natural gas odorants, and recent community-level exposure events and growing evidence of indoor natural gas leakage have raised concerns associated with natural gas odorant exposures. We conducted a scoping review of peer-reviewed scientific publications on human exposures and animal toxicological studies of natural gas odorants to assess toxicological profiles, exposure potential, health effects and regulatory guidelines associated with commonly used natural gas odorants.

**Recent Findings:**

We identified only 22 studies which met inclusion criteria for full review. Overall, there is limited evidence of both transient nonspecific health symptoms and clinically diagnosed causative neurotoxic effects associated with prolonged odorant exposures. Across seven community-level exposure events and two occupational case reports, consistent symptom patterns included: headache, ocular irritation, nose and throat irritation, respiratory complaints such as shortness of breath and asthma attacks, and skin irritation and rash. Of these, respiratory inflammation and asthma exacerbations are the most debilitating, whereas the high prevalence of ocular and dermatologic symptoms suggest a non-inhalation route of exposure.

**Summary:**

The limited evidence available raises the possibility that organosulfur odorants may pose health risks at exposures much lower than presently understood, though additional dose-response studies are needed to disentangle specific toxicologic effects from nonspecific responses to noxious organosulfur odors. Numerous recommendations are provided including more transparent and prescriptive natural gas odorant use practices.

**Supplementary Information:**

The online version contains supplementary material available at 10.1007/s40572-023-00403-w.

## Introduction

Methane (CH_4_), the primary component of natural gas, is highly combustible, yet is colorless and odorless. To aid in leak detection and promote safety, processed natural gas is intentionally odorized using a variety of organosulfur compounds. Generally, three chemical classes of organosulfur compounds are used in the natural gas industry in North America: alkyl mercaptans such as t-butyl mercaptan (TBM) defined by a terminating S-H (thiol) group; alkyl sulfides or thioethers such as dimethyl sulfide (DMS) defined by a dual-linked sulfur atom; and cyclic odorants such as tetrahydrothiophene (THT) that have a sulfur atom linked within a saturated CH ring structure. Odorants are most often added at the point where gas enters the distribution network but also are required in some high-volume transmission pipelines that intersect high population areas [1]. Overall, the effectiveness of odorizing natural gas as a safety mechanism cannot be overstated; however, no literature review has been conducted on the toxicological endpoints associated with commonly used natural gas odorant compounds and exposures in animals or humans. Given the widespread use of these various odorant chemicals in natural gas and the growing evidence of urban and indoor natural gas leakage [2–4] that can lead to prolonged human exposures, we conducted a scoping review to evaluate the scientific evidence regarding the toxicological and human health endpoints of the most widely used natural gas odorants.

This review also includes an introduction to natural gas odorant regulations and existing recommended practices for natural gas odorant chemical usage. We cross-reference reported symptomologies between documented community-level odorant exposure events to assess symptom patterns following acute to sub-chronic low-dose odorant exposures. Finally, we provide conclusions and recommendations related to improving the current understanding of natural gas odorants and human health.

### Natural gas odorant usage

In 1937, a natural gas leak at a school in New London, Texas caused an explosion that resulted in the deaths of approximately 300 students and teachers [5]. Because natural gas was not routinely odorized at that time, the leak went undetected, and students and staff were unable to evacuate before the explosion occurred. One month after the explosion, the International Association of Fire Chiefs released an investigative report citing its top prevention recommendation as the required use of “effective malodorants for detection of escaping combustible gas due to leaking equipment” [5]. As a result, a U.S. federal law mandating the odorization of natural gas was put into place and continues to form the basis of all current natural gas odorization practices.

According to Title 49, section 192.625 of the United States Code of Federal Regulations (CFR) (49 CFR **§**192.625), “a combustible gas in a distribution line must contain a natural odorant or be odorized so that at a concentration in air of one-fifth of the lower explosive limit is readily detectable by a person with a normal sense of smell” to enable evacuation of indoor spaces with dangerous natural gas concentrations. Thus, the application of odorants to natural gas are based solely upon the risk of thermal explosivity. In considering the lower explosion limit of methane, approximately 4.4% methane by volume, a natural gas leak must have enough odorant to be detected via sense of smell at one-fifth that concentration or approximately 0.88% by volume in ambient air. Notably, no other regulatory guidance is provided related to odorization standards or chemical usage. The only mention of exposure related to human health is in 49 CFR § 192.625(c)1, which states that “the odorant may not be deleterious to persons…”.

The European Union (E.U.) regulates natural gas odorant usage and standardization through a non-profit international association of 25-member organizations across 19 countries [6]. This body maintains a much more rigorous level of technical regulation, standardization, and certification of odorization practices compared to North America. For example, odorant formulas and concentrations must meet specific requirements or standards and routine sampling must be done at various points of the distribution system[6]. The majority of EU member countries have adopted minimum odorant concentrations to meet odorization requirements as opposed to olfactive or smell tests [6]. Where available, odorant concentrations are measured in gas by three different standardized analytical methods depending upon the constituent of interest [6]. Some countries have also begun utilizing admixtures of sulfur-free odorants such as ethyl acrylate [6]. While much of this review was informed by odorization practices common in North America, the EU and other parts of the world utilize many of the same sulfur-based odorants.

In practice, natural gas odorants must possess several physical and chemical characteristics to be effective for leak detection. According to industry recommended practices, odorants must have a strong and distinct odor, a high degree of chemical stability to persist in the natural gas system and the environment, a high vapor pressure to avoid condensation, a low freezing point, must not be harmful to persons, materials, or pipes, and must not create toxic combustion byproducts [7]. To achieve these properties, natural gas utilities around the world typically use blends of various organosulfur compounds, of which the most commonly reported include tert-butyl mercaptan (TBM), isopropyl mercaptan (IPM), tetrahydrothiophene (THT), n-propyl mercaptan (NPM), and dimethyl sulfide (DMS). From available information, TBM is more commonly used in North America, whereas THT is the common odorant utilized in the E.U. [6–10].

While odorants are reported to be applied to the gas stream in low concentrations (1-4 ppm [7,11]; 0-10 ppm [12]; 3-40 mg/m^3^ [6]), a study of the composition of the natural gas distribution system in Boston identified large variations in the concentration of odorants present at the point of use [13], suggesting that the concentrations at which odorants are added to natural gas and the resulting concentrations at the point of the end-user can vary greatly. In addition, physicochemical processes within pipelines can decrease odorant concentrations (i.e., odor fade) [10,14]. To address odor fade, utilities may pretreat new pipelines or add extra odorant, potentially leading to the injection of more odorant than necessary to meet the regulatory requirements [15]. However, no formal regulations address odor fade and practices may vary by operator [7,10]. Overall, variability in both natural gas odorant application and human odor detection thresholds can contribute to a wide range in odorant content at the point of the end user that can affect natural gas leak detectability and odorization efficacy [13].

Unlike the E.U., U.S. federal odorization laws do not list specific compounds or concentrations that must be used; therefore, odorant use within the U.S. has generally been regarded as proprietary. The proprietary nature of chemical odorization introduces uncertainty surrounding which odorants are used and at what concentrations. This is particularly the case for the compounds methyl mercaptan (MM) and ethyl mercaptan (EM) based on conflicting evidence of reported use. Ortiz [8] notes that methyl mercaptan is not used as a natural gas odorant due to its low molecular weight and high reactivity. This claim is supported by other publications including reports from the Agency for Toxic Substances and Disease Registry (ATSDR), the National Oceanic and Atmospheric Administration (NOAA), and the American Chemistry Council (ACC), where methyl mercaptan is explicitly considered to not be acceptable for use as a natural gas odorant [16–18]. These claims, both from industry and government sources alike, contradict similar documentation from the Department of Health and Human Services (DHHS) and other sources affirming methyl mercaptan use in U.S. natural gas systems [19–22••].

Similarly, ethyl mercaptan is not reported as a commonly used natural gas odorant [8], although some odorized natural gas materials safety data sheets (MSDS) list ethyl mercaptan as an odorant [23,24]. Ethyl mercaptan, however, is commonly used as a natural gas odorant in Romania and for liquified petroleum gas (LPG) (e.g., propane) [6,8,20,25–29]. Notably, Hazardous Materials Regulations in the U.S. require LPG to be effectively odorized using one pound of ethyl mercaptan per 10,000 gallons for transportation [28,30], though in practice 1.5-2.5 pounds may sometimes be used [27]. Overall, methyl mercaptan and ethyl mercaptan appear to be used only sparingly if at all as natural gas odorants in the U.S. [7–10]. Nonetheless, the literature on the acute health impacts of methyl- and ethyl mercaptan have been thoroughly reviewed by the National Research Council [31] and—in the case of methyl mercaptan—by the ATSDR [17,32] leading to the development of exposure guidelines for these compounds (Table 1). For these reasons, methyl- and ethyl mercaptan were excluded from this review. Thus, this review focuses on the most common and consistently reported natural gas odorants—TBM, IPM, THT, NPM, and DMS [7–10].

**Table 1:** Physical and chemical properties and various regulatory limits of common natural gas odorants. Note that ESLs from the TCEQ are interim guidelines that have not been finalized. Odorants are listed by frequency of use within the natural gas system and note that blends of multiple odorants are often utilized. Methyl mercaptan and ethyl mercaptan are included for reference only and are not regularly reported as used in natural gas but have been used as surrogate mercaptan compounds in accidental release events [22••].

Odorant	CASRN	Odor Threshold (ppb)	Oxidation Stability	Freezing Point (°C)	Organic Carbon-Water Partition Coefficient (Koc)	Vapor Pressure (mmHg at 25°C)	Vapor Density (Air = 1)	OSHA PEL^*a*^ (ppm) 8 hr TWA	NIOSH REL^*a*^ (ppm) 15 min TWA	ACGIH TLV^*b*^ (ppm) 8 hr TWA	MAK (ppm) 40 hr/week^*c*^	TCEQ ESL Short-term (8-hour) (ppb)	TCEQ ESL Long-term (1 year) (ppb)	Commonly Used Natural Gas Odorant
Tert-butyl mercaptan (TBM)	75-66-1	0.029^***d***^-0.9^***e***^	High^***f***^	-0.5^***g***^	49^***g***^	181^***g***^	3.1^***g***^	**-**^***h***^	**-**^***i***^	**-**	**-**	4.9^***j***^	0.49^***j***^	X
Isopropyl mercaptan (IPM)	75-33-2	0.0008^***d***^-0.006^***k***^	High^***f***^	-131^***l***^	35^***l***^	277^***l***^	2.610^***m***^	**-**	**-**	**-**	**-**	5.8 ^***j***^	0.58 ^***j***^	X
Tetrahydrothiophene (THT)	110-01-0	0.62^***d***^-1^***n***^	Good^***f***^	-96^***n***^	**-**	18.4^***n***^	3.05^***n***^	**-**	**-**	**-**	50	500^***o***^	50^***o***^	X
Dimethyl sulfide (DMS)	75-18-3	0.12^***k***^-63^***p***^	Good^***f***^	-98^***p***^	6.3^***p***^	502^***p***^	2.14^***p***^	**-**	**-**	10	**-**	100^***o***^	10^***o***^	X
n-Propyl mercaptan (NPM)	107-03-9	0.013^***d***^-1.6^***q***^	Low^***f***^	-113^***q***^	230^***q***^	154^***q***^	2.63^***q***^	**-**	0.5	**-**	**-**	5^***o***^	0.5^***o***^	X
Methyl mercaptan (MM)	74-93-1	0.0002^***d***^-19^***k***^	-	-123^*r*^	13^*r*^	1510^*r*^	1.66^*r*^	10	0.5	0.5	0.5	5^***o***^	0.5^***o***^	
Ethyl mercaptan (EM)	75-08-1	0.0087^***d***^-1^*s*^	-	-148^*t*^	22^*t*^	529^*t*^	2.14^*t*^	10	0.5	0.5	0.5	5^***o***^	0.5^***o***^	

a.“NIOSH Pocket Guide to Chemical Hazards,” 2007; b. OSHA Occupational Chemical Database, 2020; c.DFG Deutsche Forschungsgemeinschaft, 2015 d. Nagata 2003; e. Moschandreas et al., 1982; f. Tenkrat et al., 2010; g.“PubChem: 2-Methyl-2-propanethiol,” n.d.; h. OSHA 8 hr TWA PEL for n-butyl mercaptan is 10 ppm; i. NIOSH 15 min TWA REL for n-butyl mercaptan is 0.5 ppm; j. Health-based ESL using surrogate compound “mercaptan, not otherwise specified” (interim ESL); k. Clanton and Schmidt, 2000; l. PubChem: 2-Propanethiol,” n.d.; m. Luebke, 2018; n. “PubChem: Tetrahydrothiophene,” n.d.; o. Health-based ESL using occupational exposure guidance levels (interim ESL); p. “PubChem: Dimethyl sulfide,” n.d.; q.“PubChem: 1-Propanethiol,” n.d.; r “PubChem: Methanethiol,” n.d.; s. Leonardos, Kendall, & Barnard, 1969; t. “PubChem: Ethanethiol,” n.d.

### Natural gas odorant exposure guidelines

Various international regulatory bodies set general and workplace exposure guidelines to protect human health based upon available scientific evidence. For example, the US Environmental Protection Agency (USEPA) provides Reference Concentrations (RfC) or non-hazardous inhalation exposure levels for numerous chemicals.). Similarly, the California Office of Environmental Health Hazard Assessment (OEHHA) sets acute and chronic reference exposure levels for numerous compounds intended for use in community health assessments. For occupational settings, occupational exposure limits can be set by the Occupational Health and Safety Administration (OSHA), the National Institute for Occupational Safety and Health (NIOSH), and the American Conference of Governmental Industrial Hygienists (ACGIH; see Table 1). OSHA can set permissible exposure limits (PELs) limiting the concentration of a chemical substance or physical agent individuals may be occupationally exposed to over an 8-hour period (the typical length of a work shift) [33]. Similarly, NIOSH and ACGIH set recommended exposure limits (RELs) and threshold limit values (TLVs), respectively, for exposure to hazardous substances or conditions in the workplace [34]. Other relevant occupational exposure limits can be informed by the German Research Foundation (DFG, Deutsche Forschungsgemeinschaft) Commission for the Investigation of Health Hazards of Chemical Compounds in the Work Area, better known as the MAK Commission [35].

Overall, community-level, and occupational exposure limits are lacking for commonly used odorants or rely upon surrogate compounds (e.g., n-butyl-mercaptan as a surrogate for tert-butyl-mercaptan) (Table 1). Although THT, DMS, and NPM have at least one occupational exposure limit guideline, it is important to note that these limits are inappropriate for broader community exposure guidance due to different exposure scenarios and varying susceptibilities within the broader population. Outside of the regulatory agencies above, the Texas Commission on Environmental Quality (TCEQ) recently developed interim short-term and long-term effects screening levels (ESLs) for commonly used natural gas odorants with the intent to protect human health in the general public as well as to prevent nuisance odors and harmful effects in vegetation. However, these ESLs have not been finalized and long-term ESLs are based on the occupational benchmarks where available; or in case of TBM and IPM, are based on unspecified surrogate compounds [36,37]. To date, it appears that these ESLs have only been used for air permit requirement purposes. And while these ESLs are not ambient air standards, they do “represent concentrations in outdoor air below which adverse effects on health or welfare are not expected” [37,38]. Until finalized however, it remains unclear how these interim ESLs were developed or what surrogate compounds were used.

## Methods

We conducted a scoping literature review by searching PubMed and Web of Science databases for literature related to the health hazards, risks, and impacts of DMS, THT, TBM, NPM, and IPM exposure. The search was conducted on February 21, 2022; a complete list of search terms can be found in Online Resource 1. Resulting articles were initially screened by title and then by abstract. We included titles that indicated that the article was an in vivo (i.e., human or animal) or in vitro study of an odorant of interest. If the odorants were not explicitly named in the title, but the title indicated it was a human, animal, or cell culture-based study, then abstracts were screened to determine the odorants studied. The abstracts were screened according to the following inclusion criteria:

1. English language
2. Odorant of interest
3. Original study
4. External exposure to odorants of interest (as opposed to biological breakdown product or modeling study)
5. Includes biological endpoint

Studies that included co-exposure to other sulfur compounds were included during our screening process. In addition to the primary PubMed and Web of Science searches, we also identified relevant studies that were cited by the articles included in the final review. When relevant citations were inaccessible (due to either language, age, or being unpublished), summaries from the citing article were included herein.

## Results

Our search yielded a total of 1,585 unique articles, of which 119 remained after titles were screened for relevance. After abstracts were screened for our inclusion criteria and additional studies were included, a total of 22 articles were identified, of which ten were human exposure studies, eleven were animal studies, and one was a cell culture study. Despite a long history of efficacy as a leak detection system, studies of organosulfur exposure in humans have generally been limited to accident-related community exposure events and low sample-size case reports in occupational settings following chemical spills. One prominent community exposure event at the Aliso Canyon underground gas storage facility in Porter Ranch, California was not included in our primary literature search or article citations but was included in our review given its high-profile nature and motivation to perform this review. The results of our literature search, screening process, and a complete list of articles included in our review are provided in Online Resource 1. We discuss the results of these studies below first by specific odorant and then from a multi-pollutant perspective.

### Dimethyl sulfide (DMS)

#### Human studies

One case of occupational exposure to DMS occurred in a paper manufacturing plant in Japan when two men entered a storage tank and immediately collapsed. One was deceased when found and the other died one and a half days later. Though MM and DMS were both potentially present, blood analysis revealed DMS as the primary inhaled gas [39••]. Autopsy results and accompanying animal investigations suggest that the cause of death was due to asphyxia due to displacement of atmospheric oxygen by the DMS gas [39••]. Other human exposure studies of DMS in the literature have concurrent exposures to other sulfur compounds (e.g., hydrogen sulfide (H_2_S), MM, etc.) [40•–42•] that were not disentangled and are subsequently discussed below with other multi-pollutant exposure events.

#### Animal studies

The majority of animal studies were acute exposure studies that used death as their primary endpoint. One study that focused on behavioral changes in rats found that a 0.5g/kg dose of DMS resulted in decreased motor activity when administered both orally and intraperitoneally, as well as reduced body temperature when placed in a 5°C environment after intraperitoneal administration of DMS [43]. Another study that reported symptoms of acute exposure in rats found that DMS increased secretions from the eyes and nose as well as voluntary and respiratory muscle paralysis starting at 0.56% v/v DMS [44]. DMS resulted in mortality in 15 minutes at 5% v/v [44]. In another acute exposure experiment in rats, intraperitoneal DMS injection produced a median effective dose (ED_50_) for coma of 817 mg/kg (dose producing coma in 50% of rats) and median lethal dose (LD_50_) of 537 mg/kg (dose producing death in 50% of rats). Rats at higher doses also displayed ataxia, either alone or accompanied by subsequent loss of the righting reflex as well as an increased heart rate and respiration rate [45]. Another acute inhalation exposure study in rats found a 4-hour median lethal concentration (LC_50_) of 40,250 ppm [46]. Similarly, an acute inhalation study in mice found that 100% of mice became immobile within one minute and died within eight minutes of exposure above 6.8±1.3% v/v DMS [39••]. However, another study that looked at short-term oral exposure to smaller doses (up to 250 mg/kg/day for 14 weeks—equivalent to an intake of 17.5 g/day by a 70 kg adult) did not report any adverse effects for all dose levels [47,48]. It should be reiterated that concentrations of odorants used in these animal studies are significantly greater than would be expected from a distribution-grade natural gas leak, and not all controlled studies assessed exposures through inhalation. For example, the maximum DMS concentration reported in the E.U. gas system was 10 mg/m^3^ for 100% natural gas, indicating that exposure to a small gas leak at 0.1% gas would have an equivalent maximum DMS concentration of 0.01 mg/m^3^.

A biochemical pathway for DMS toxicity has also been suggested based upon its ability to inhibit rat hepatic dimethylnitrosamine demethylase [49]. Dimethylnitrosamine demethylase is a mixed function oxidase that metabolizes a large array of lipophilic drugs and endobiotics [50,51]. In another study, DMS exhibited a small inhibitory effect on rat mitochondrial respiration [52]. In another study, DMS served as a functional substrate for methionine sulfoxide reductase A, a key antioxidant enzyme, and reduced oxidative stress and increased lifespan in *C. elegans* (roundworms) and *Drosphila* (fruit flies) [53]. While the impact of DMS in human cells is not elucidated in these studies, they suggest possible biological pathways for DMS exposure to influence human biology and alter metabolism of select pharmaceuticals.

### tert-Butyl mercaptan (TBM)

#### Human studies

In 2008, TBM intended for natural gas odorization leaked from a storage tank following a lightning strike at the Gulf South natural gas pumping station in Prichard, Alabama [54]. The health effects from airborne exposure to the mercaptan were not investigated until 2012, when a contaminated spring was discovered. Ambient TBM concentrations were measured only at the outfall of the spring reaching a maximum of 230 ppb. While no other ambient sampling took place, odor complaints of nearby residents were documented over a 6-month period [54]. 37% of the study population living within a 2-mile radius sought medical care for perceived odor-related symptoms, with self-reported symptoms including nausea, dizziness, headaches, general weakness, nasal congestion, sinus infection, shortness of breath, cough, wheezing, asthma exacerbation, skin irritation, and eye, nose and throat irritation among other complaints. Odor severity and the occurrence of self-reported symptoms were greater among residents living within a one-mile radius as compared to residents living within a two-mile radius [54]. While definitive exposure levels could not be determined, concentrations were likely significantly lower in the 2-mile radius compared to the source (230 ppb) based solely upon general dispersion and dilution dynamics. Overall, this study provides some evidence that long-term exposure to TBM at very low concentrations can induce psychological, gastrointestinal, dermal, cardiovascular, and respiratory symptoms that have the potential to exacerbate underlying health conditions such as asthma.

#### Animal studies

In an animal study, TBM was classified as “practically non-toxic" due to its relatively high 15-day post-inhalation LC_50_ of 22,200 ppm/4h in rats and 16,500 ppm/4h in mice [55]. However, concentrations of TBM at near lethal levels resulted in muscle weakness, ataxia, increased respiration and restlessness, partial skeletal muscle paralysis, sedation, and cyanosis in both rats and mice [55]. TBM exposure also resulted in mucous membrane irritation and increased respiration, similar to the symptoms reported in the human exposure event in the Eight Mile community of Prichard, Alabama [54,55].

### Isopropyl mercaptan (IPM)

Our review did not find any IPM human exposure or animal studies. The only study investigating potential health effects of IPM was a cell culture study that found that the reaction products of selenite with IPM (and other thiols) inhibit amino acid incorporation and protein synthesis [56]. While interesting, this study does not provide information on the toxicity of IPM on its own. The paucity of literature on IPM is particularly notable given its relatively frequent use as a natural gas odorant (second most common) [7–9].

### n-Propyl mercaptan (NPM)

#### Human studies

There have been two major community NPM exposure events, both of which resulted from the degradation of ethoprop, an organophosphate acetylcholinesterase inhibitor insecticide [22••,^57^••]. NPM is both a manufactured precursor and a degradation product of ethoprop, and is slowly released into the environment following the application of the insecticide. The first exposure event occurred in 1989 in Dorris, California after ethoprop was applied to 145 acres of potato fields [57••]. Soon after its application, nearby residents began complaining of a number of health effects including headache (odds ratio [OR] = 5.08), diarrhea (OR = 3.80), runny nose (OR = 5.31), nausea (OR = 3.39), vomiting (OR = 1.86), sore throat (OR = 3.58), burning/itching eyes (OR = 5.64), fever (OR = 3.59), difficulty breathing (OR = 3.44), hay fever attacks (OR = 3.50), and asthma attacks (OR = 6.0) [57••]. Survey data also noted that the stronger the perceived odor and the longer the exposure duration, the greater the number of health effects reported [57••]. Ames and Stratton [57••] considered direct exposure to ethoprop unlikely because the compound was incorporated into the soil and therefore attributed observed symptoms to NPM exposure, though no in situ air monitoring was performed.

The second NPM exposure event occurred in 2006 when a shipment of ethoprop degraded at a wastewater treatment plant in Fairburn, Georgia and resulted in the release of NPM into the air [22••]. Air samples taken during the incident resulted in non-detects (albeit with a 0.5 ppm limit of detection) even though residents reported the odor lingering for several weeks [22••]. During the event, the ATSDR recommended an outdoor NPM action level—the concentration below which no permanent health effects are expected to occur—of 0.5 ppm based on chemically functional similarities between NPM and methyl mercaptan. Notably, the 0.5 ppm action level is two to four orders of magnitude greater than the NPM odor detection threshold (0.013-1.6 ppb [58,59]; see Table 1) and equal to both the limit of detection for the monitoring instrument deployed and the NIOSH 15 min recommend exposure limit, which are typically not appropriate for general population exposures [22••]. Symptoms reported were similar to those in the Dorris, California exposure event and included headache (74%), burning eyes (58%), cough/sore throat (54%), nausea/vomiting (49%), and difficulty breathing (45%). 41% of people reported other symptoms such as chest congestion or tightness, skin irritation, diarrhea, and fatigue, with most people reporting multiple symptoms [22••]. Despite numerous reports of symptoms, it was concluded that the site was “not a public health hazard because all air samples were below the action level and long-term health impacts were not expected” [22••]. Nonetheless, both studies must be interpreted with caution due to significant overlap between the reported symptoms and those caused directly by ethoprop exposure and other similar acetylcholinesterase inhibitors [22••,^57^••,^60^].

#### Animal studies

Analysis of medical records of dogs and cats from the Fairburn, Georgia exposure event showed an increase in frequency of gastrointestinal symptoms in dogs and eye inflammation in cats following the chemical release; however, both of these clusters of symptoms were reported in geographically separate locations [22••,^61^]. Furthermore, the researchers did not know the home range of each pet, so exposure to NPM of each animal could not be estimated [61]. Despite the limitations of the companion animal study, the results complement the evidence from the human exposure events that NPM is associated with symptoms of nausea/vomiting, diarrhea, and eye irritation [22••,^61^].

The same animal toxicological study that investigated TBM also looked at NPM and calculated 15-day post-inhalation LC_50_ for rats (7,300 ppm/4h) and for mice (4,010 ppm/4h) [55]. The authors classified NPM as slightly toxic. The authors also reported that NPM inhalation at near-lethal concentrations for both rats and mice resulted in similar symptoms as TBM (muscle weakness, ataxia, increased respiration and restlessness, partial skeletal muscle paralysis, sedation, cyanosis, and mucous membrane irritation) [55].

In another study cited by Fairchild and Stokinger [55], but inaccessible, a 4-hour rat LD_50_ of 4,100 ppm was found for a thiol mixture of 24% NPM, 55% butyl mercaptan, and 21% amyl mercaptan [62]. Inhalation of thiols was considered a moderately severe hazard due to resulting pneumonitis and tracheitis [62].

### Tetrahydrothiophene (THT)

#### Human studies

Two reports of human exposure of tetrahydrothiophene (THT) have been documented with overlapping symptoms including headache, nausea, and shortness of breath [63•,^64^••]. The first report was for two separate occupational exposures—a 73-year-old (Case A) and a 53-year-old (Case B)—both of whom were natural gas odorization workers employed at the same facility. There were no direct measurements of THT during the relevant occupational exposures settings, but THT concentration during odorization was estimated to be between 3-4 ml/m^3^ as determined by a technical consulting service retained by the statutory accident insurance institution [64••]. The 73-year-old male performed odorization activities one to three times per week from 1968-1971, with each event lasting approximately an hour. Odorization activities took place in a small chamber with no ventilation or personal protective equipment. The 53-year-old performed odorization tasks from 1970-1982 in the same facility, over which time improvements to THT containers and personal protective equipment eventually likely limited exposure. Both men reported symptoms of nausea, vomiting, headache, mucosa irritation, rhinitis, and difficulty breathing during work exposure [64••]. Both men also reported acute dermal irritation presenting with eczematous, and reddened scaly skin alterations following acute exposure. Their symptoms gradually worsened over the course of their odorizing activities, with some persisting during non-work hours. After cessation of odorizing activities, both Case A and Case B showed improvements; however, chronic shortness of breath and respiratory impairment persisted. Neither had a pertinent family history, nor evidence that any of their medical problems were pre-existing conditions, though Case B did report a history of smoking. Nonetheless, it was concluded by the study authors that their chronic obstructive pulmonary disease (COPD) was a direct result of chronic occupational exposure to THT and as a result, both patients’ diagnoses were accepted by their respective insurance institutions and were granted compensation. Study authors concluded that in the light of severe central nervous system disorders observed in animal studies, the nausea, vomiting, headaches, loss of appetite reported by the exposed workers likely represent transient symptoms. Both the improvement of symptoms after cessation of THT exposure and the temporal relationship between long-term THT exposure and the development of severe respiratory symptoms suggested a causative association.

The second exposure event occurred in Hong Kong, when 70 adolescent-aged school children from two nearby schools were taken to hospitals for assessment and observation associated with exposure to a THT spill on a nearby barge [63•]. Reported symptoms included headache, dizziness, nausea, and shortness of breath, with improvements observed after leaving the site of exposure [63•]. Ambient concentrations of THT during the exposure event were not reported, and unfortunately, no other details were made available.

#### Animal studies

No animal studies met our review inclusion criteria; however, Baur and Bittner [64••] cited multiple unpublished or inaccessible studies as well as non-English studies related to animal toxicity and THT. Given the importance of this weight of evidence in supporting their causal inference claims, we included a summary of their review below.

Following acute inhalation exposures, animals have presented a wide range of effects including general dysfunction of the central nervous system (hyperactivity, motor hyperreflexia, sedation, narcosis), irritation of eye and nose mucosa, dyspnea associated with hyperinflation and severe lung damage, peripheral vasodilation, and, when combined with adrenalin, cardiac arrhythmia, and bradycardia [65–72]. Chronic inhalation tests with mice and rats showed lacrimation, hypersalivation, liver dysfunctions and behavior disorders (hyperactivity, temporary aggression followed by depression) [70,73]. A study of the effect of THT exposure on pregnant rats found a no observed adverse effect concentration (NOAEC) of 234 ml/m^3^ for maternal toxicity (nose, eye, and skin irritation, agitation) and of 1,910 ml/m^3^ for adverse fetal effects [74]. Oral uptake additionally induced damage to liver, kidneys, and intestines [66].

### Multi-pollutant exposure events

In addition to single odorant exposure events, there are multiple health effects studies where exposure to multiple sulfur-containing compounds occurred. A cross-sectional hygienic survey conducted in pulp mills detected DMS (0-15ppm), MM (0-15ppm), H_2_S (0-20 ppm), and dimethyl disulfide (DMDS) (0-1.5ppm); workers exposed to these chemicals reported chronic headaches more often than controls [42•]. Although workers in the exposed group also reported a lack of mental concentration, restlessness, and a lack of vigor more frequently than the control group, the differences were not statistically significant [42•]. One study looked at the combined health effects of various sulfur pollutants released from pulp mills, including DMS, H_2_S, MM, DMDS, and sulfur dioxide on adults living in areas polluted with these compounds [41•]. Occurrence of cough, headache, and eye and nasal symptoms were higher among adults living in moderately polluted (4 km from one pulp mill) and severely polluted (0.5 km from one pulp mill and 3 km from another) communities as compared to those living in non-polluted communities (>100 km away) [41•]. In a similar study, a survey questionnaire of residents living near a cellulose paper plant that emitted DMS, H_2_S, MM, and other unspecified air pollutants found that 27% of residents who perceived the odor reported headache, 19% reported disturbed sleep, and 30% experience nausea, vomiting, stomach discomfort, and palpitations [40•]. Each of these studies shared headache as a symptom; however, the extent of which reported symptoms can be attributed to exposure to odorants, as opposed to exposure to other compounds such as H_2_S, is unknown.

One additional multi-pollutant exposure event that was not part of the primary literature search is worth noting: the Aliso Canyon underground natural gas storage facility blowout in the town of Porter Ranch in Southern California [75]. During the 118 day leak from October 2015 to February 2016, the Los Angeles County Department of Public Health received over 700 health complaints associated with exposure to emissions from the leaking well [76]. Southern California Gas Company, the operator of the Aliso Canyon facility, disclosed that a mixture of TBM and THT were added to the natural gas stored at Aliso Canyon [75,77]. During the leak, a projected 81% (6,278) of households in the nearby Porter Ranch reported symptoms including eye, nose and or throat irritation, headache/migraine, respiratory complaints (e.g., cough, tightness in the chest, difficulty breathing, shortness of breath, worsening of asthma or COPD), stress, dizziness lightheadedness, nausea vomiting, nosebleeds, skin rash/irritated skin, diarrhea, and fever [75,78••]. This value may be an underestimate as affected residents were temporarily relocated over the course of the leak [78••,^79^]. OEHHA stated that self-reported health symptoms, with the exception of fever, were attributable to odorants in natural gas [80,81]; however, no measurements of mercaptans exceeded the 5 ppb detection limits of monitoring equipment (5 – 172 times greater than known odorant thresholds) [78••,^80^]. Similarly, the Los Angeles County Department of Public Health stated that exposure to concentrations of mercaptans below monitoring detection limits could cause symptoms reported by residents including eye, nose and throat irritation, coughing, shortness of breath, nausea, headaches, and dizziness [77]. Symptoms from exposure to mercaptans are dependent on frequency and duration of exposure and are not expected to result in long-term health effects [77].

Numerous other confounding factors also likely contributed to reported symptoms following the Aliso Canyon accident. In addition to TBM and THT, there were a number of co-pollutants emitted during the release that may have also contributed to ambient odors (e.g., H_2_S, benzene, petroleum hydrocarbons, well-control chemicals, etc.) and introduced independent health effects or synergistic effects that exacerbated or compounded health effects consistent with mercaptan exposure [75]. Nonetheless, the attribution of symptoms to odorant exposures at concentrations below ambient monitoring instrument detection limits (~5 ppbv), yet above human odor detection thresholds (e.g., <0.1 ppbv; see Table 1) reflects the malodor potency of this class of compound. This potency gives organosulfur compounds its utility as a malodorant in natural gas systems. However, this potency becomes a liability during accidental release events whereby very low-level chronic exposures (e.g., >0.1ppbv) can induce a wide range of debilitating symptoms on the public in part due to the wide range of susceptibilities to noxious odors. These types of widespread exposure events can also trigger sympathetic nervous system responses (stress response), and henceforth condition a subject to associate odors and specific harms, such as asthma exacerbation [82].

### Summary of symptoms prevalence associated with organosulfur exposure

Frequency and odds ratios of symptoms reported in each of the seven community-level exposure events are presented in Table 2; however, not all symptoms were measured or surveyed for each event [22••,^54^,^57^••,^83^]. Ideally, self-reported health effects studies should have reported odds ratios or similar risk indicators to better isolate effects from exposures. Unfortunately, each study employed a slightly different exposure assessment survey method, and some studies were confounded by the presence of multiple odorant compounds or other non-sulfur pollutants. Moreover, no study formally measured ambient concentrations (or utilized sampling protocols at detectable limits that were significantly greater than typical odorant detection thresholds) near affected populations. Nonetheless, some trends were observed across these events including symptom prevalence within exposed groups.

**Table 2:** Frequency or odds ratio of reported health complaints for single and multi-pollutant community odorant exposure events. List of symptoms is non-exhaustive. NA = not evaluated in the study

	Single Pollutant Community Exposure Studies			Multiple Pollutant Community Exposure Studies			
Reference	Behbod et al., 2014 [54]	U.S. Department of Health and Human Services, 2008 [22••]	Ames and Stratton, 1991 [57••]	Los Angeles County Department of Public Health, 2016 [78••]	Georgieff and Turnovska, 1999 [40•]	Jaakkola et al. 1990 [41•]	Kangas, 1984 [42•]
Location	Eight Mile, AL	Fairburn, GA	Dorris, CA	Aliso Canyon Underground Gas Storage Facility, Porter Ranch, CA	Bulgaria	Finland	Finland
Exposure source	Contaminated spring	Wastewater treatment facility	Treated agricultural field	Natural gas storage facility	Cellulose paper plant	Pulp mills	Pulp mills
Compounds exposed to	TBM	NPM	NPM	TBM, THT	DMS, MM, H_2_S, etc.	DMS, MM, H_2_S, DMDS, sulfur dioxide	DMS, MM, H_2_S, DMDS, sulfur dioxide
Reporting value	Percentage of individual respondents (n = 204)	Percentage of individual respondents (n = 622)	*Odds ratio*	Percentage of households reporting (n = 210)	Percentage of individuals who perceived the odor (n = 480)	*Odds ratio*	Percentage of exposed individuals (n = 81)
Headache (various units)	63.2	74.3	*5.08*	71.8	26.8	*1.6-2.2*	12.3
Eye, nasal and/or throat irritation (various units)	40.2 (eye) 46.6 (throat); 54.4 (nasal); 56.4 (cough)	57.7 (burning eyes); 53.9 (cough/sore throat)	*3.58 (sore throat); 5.31 (runny nose); 5.64 (burning/itching eyes)*	73.9	51.9	*11.7-11.8 (eyes); 2.0-2.2 (nasal); 1.9-3.1 (cough)*	NA
Respiratory complaint (various units)	46.1 (sinus); 37.8 (difficulty breathing); 15.7 (asthma exacerbation)	45.5 (difficulty breathing)	*6.0 (asthma attacks); 3.5 (hay fever attacks); 3.44 (difficulty breathing)*	67	NA	*1.6-2.4 (breathlessness or wheezing)*	NA
Skin rash or irritation (various units)	37.3	9.2	*2.43*	46.1	53.8	*NA*	NA
Dizziness (various units)	36.8	5.3	*NA*	59.9	NA	*NA*	NA
Nausea or vomiting (various units)	28.9	48.7	*1.86 (vomiting); 3.39 (nausea)*	54.4	30.2	*NA*	NA
Diarrhea (various units)	23.5	6.6	*3.80*	27	NA	*NA*	NA
Cardiovascular (various units)	27.5 (chest pain); 23.5 (irregular heart beat); 28.9 (exacerbation of hypertension)	.5 (rapid pulse, elevated blood pressure)	*NA*	NA	NA	*NA*	NA
Trouble sleeping (various units)	52	NA	*NA*	NA	18.8	*NA*	NA
Nosebleed (various units)	NA	13.7	*NA*	30.9	NA	*NA*	NA

We ranked symptom prevalence across the seven community-level exposure events as shown in Table 2. Overall, headache was the most reported symptom and was also the only symptom measured and reported in all community exposure studies. Headache was most prevalent for two of the events, and second most reported for two other events. Mucous membrane (nose, throat, and eye) irritation was the next most prevalent set of symptoms. Notably, eye irritation was always more prevalent than nose and throat irritation and was the most prevalent symptom reported in association with the Finnish pulp mill exposure event [41•]. In many cases, respiratory complaints (i.e., difficulty breathing) were at similar prevalence levels to eye, nose, and throat irritation. Asthma attacks resulted in the highest odds ratio related to NPM exposure during the event in Dorris, California—higher than any other symptom reported during that event. Asthma exacerbations were only formally measured in one other event, the TBM exposure event in Eight Mile, Alabama, with only 15% of respondents reporting asthma exacerbations but exposures were to TBM not NPM. The other NPM exposure event in Fairburn, Georgia also noted a significant burden of breathing difficulty (45.5% prevalence), but ultimately it is difficult to parse the severity of respiratory effects across study surveys. Although the studies of NPM and TBM did not directly measure exposure concentrations—instead using proximity as a proxy for exposure—the study of chronic occupational exposure of THT was estimated at 3-4 ml/m^3^ (3-4 ppm) which is below the occupational German maximum workplace exposure limit (50 ppm assuming a 40-hour work week [35]), but well above the interim TCEQ long term ESL of 50 ppb [64••]. These limited studies suggest that low-level chronic exposures can contribute to various degrees of respiratory tract inflammation, though no studies have conducted long-term follow-ups from any of the community exposure events. Other symptoms such as cardiovascular conditions, nosebleeds, and trouble sleeping were not consistently reported across exposure events therefore limiting the generalizability and apportionment of these symptoms to odorant exposures.

Notably, dermal irritation or rashes were relatively prevalent across the seven events and were observed in controlled studies as well. In two of the events, nearly half of respondents reported some degree of dermal irritation. Of the symptoms reported, the high prevalence of skin and eye irritation provides evidence that organosulfur compounds may manifest a direct toxicological effect through cutaneous absorption rather than through inhalation. While the etiology of skin irritation therein is ultimately unclear (e.g., contact dermatitis, atopic dermatitis, or other inflammatory response), previous research has shown associations between atopic dermatitis and certain air pollutants with a hypothesized mechanism whereby reactive oxygen species are generated by environmental exposures that cause damage to proteins in the stratum corneum [84].

Acute symptoms reported in case studies of known and isolated THT exposures included headache, dizziness, nausea, vomiting, headache, skin and mucous membrane irritation, and difficulty breathing and provide additional evidence of a consistent collection of symptoms from acute THT exposure [63•,^64^••]. Bauer and Bittner [64••] established a causal link between these symptoms and THT exposure noting that exposed workers experienced transient neurotoxic effects as determined by symptom cessation following exposure attenuation [64••]. Most notably, THT is only odorant that has a documented causal link between long-term exposure and clinically diagnosed COPD [64••]. The causal link was supported by three contributing pieces of evidence: severe central nervous system disorders observed in animal studies, the temporal relation between exposure and occurrence of respiratory symptoms as well as the initial improvement after exposure termination, and the fact that both subjects developed COPD after long-term high THT contact.

No community exposure events were found for IPM, and likewise very little human, animal, or cell studies were found for IPM. Symptoms related to DMS exposure were difficult to isolate given the nature of the multi-pollutant studies and the presence of MM and H_2_S, which are known to cause symptoms similar to other odorants including headache, dizziness, nausea, vomiting, mucous membrane irritation, and difficulty breathing [19,85,86]. Furthermore, no symptoms were reported for the DMS occupational exposure event that resulted in the death of two workers [39••]. However, animal studies suggest that DMS may cause symptoms similar to those reported for other odorants [44]. In general, reports from these seven community-level exposure events and case studies show a consistent symptomatology associated with low dose sub-acute and chronic exposures to TBM, NPM, THT, and DMS with few clear differentiating trends between individual compounds and symptoms.

## Discussion

For each of the commonly used natural gas odorants investigated (except IPM), this review indicates that odorants can induce a range of adverse symptoms; however, very little information exists related to associated exposure concentrations, or symptom etiology. Few studies have measured *in situ* ambient concentrations of odorants during exposure events; available concentration data ranges from below monitored detection limits for community-scale events to very high doses for occupational settings and controlled animal studies. Importantly, monitoring equipment deployed during accidental release events has limits of detection orders of magnitude greater (5-500 ppb, depending on the study) than typical human olfactory thresholds (typically <0.1 ppb), rendering them unable to detect airborne concentrations at levels approaching human olfactory detection. This further obfuscates differentiation between olfaction and sensory irritation or any potential toxicological effects.

Consistency in symptom constellations does not imply definitive symptom etiologies, and could be result of specific toxicological processes, transient malodor responses, and/or an overreactive sympathetic nervous system response. Malodors for example, such as those produced by organosulfur compounds, can negatively impact mood, stress levels, and cognition, as well as elicit symptoms including headaches, dizziness, nausea, vomiting, sleep disorders, and irritation of the eyes, nose, and throat [82]. These symptoms may arise from a sympathetic nervous system response (stress response), past experience with the odor, and/or conditioning between an odor and specific harms, such as asthma flares [82]. As noted by Behbod et al., [54] because mercaptans have extremely low odor thresholds, their disagreeable odors alone could be responsible for the genesis of the reported symptoms, as opposed to a specific toxicological effect. In line with previous recommendations, studies are still needed to better elucidate the distinction between transient malodor responses and/or generalized stress responses and any toxicological effects of odorant exposure.

Most notably, THT is only odorant with evidence linking long-term exposure and clinically diagnosed COPD, though this study involved only two cases from a single natural gas odorization facility [64••]. Nonetheless, Baur and Bittner [64••] determined a causal link between THT exposure and symptom presentation noting that exposed workers experienced transient neurotoxic effects as determined by symptom cessation following exposure attenuation [64••]. The estimated ambient THT level within the occupational setting was 3-4 ml/m^3^ (3-4 ppm)—an exposure level significantly lower than the 40hr/week MAK occupational standard of 50 ppm (Table 1) [35]. Study authors recommended a more detailed investigation and evaluation of the literature related to THT to support the development of a health based TLV, which has not been stipulated in the U.S. or elsewhere [64••].

Although odorant exposures to the general population can result from major natural gas release events, more significant exposures may result from smaller leaks in downstream distribution infrastructure and behind-the-meter (i.e., within buildings). In the indoor environment, natural gas leaks from pipes, pilot lights, and a variety of appliances including stoves, space heaters, and water heaters have been characterized. Leaks can occur with during both appliance (e.g., gas stove) operation and while appliances are off [3,13,87,88]. Sargent et al., [2] found that an estimated 2.5 ± 0.5% of natural gas entering the Boston region is leaked to the atmosphere, noting that emissions are correlated with seasonal end-use consumption (i.e., increased use during the winter heating season) implying that emissions may be significantly underestimated in indoor spaces from leaking residential end-use appliances. Given the implications posed by Sargent et al., [2] that indoor gas leaks may be much more common than previously understood, multiple questions arise related to odorization of natural gas: 1) are chronic, low dose exposures to natural gas odorants more prevalent than previously understood; 2) do smaller leaks contain too little odorant to be detected due to too little odorant to reach general odorant thresholds, either from odor fade, odor masking, olfactory dysfunction, or olfactory habituation; 3) do many of these leaks contain enough odorant to be detected, but are ignored for various reasons (i.e., not believed to be serious enough to seek a fix, financial or other constraints, etc.); 4) what are the effects of multiple odorants in natural gas on odorant detection and health effects; and, 5) what are the potential health effects of odorant degradation bi-products in residential natural gas supplies?

### Study limitations

Our review was limited to organosulfur compounds that have documented use as natural gas odorants and English-written peer-reviewed studies of associated health effects. Due to the lack of transparency in odorant disclosure, odorants other than the five we identified may be used in natural gas but were not represented in our review. We also did not include gray literature, government reports, industry reports, or toxicity assessments beyond the exposure events from the loss of containment event at the Aliso Canyon Underground Gas Storage Facility in Porter Ranch, California and at the wastewater treatment facility in Fairburn, Georgia. The number of toxicological- and health-related studies in our review may therefore be under representative due to our search strategy, and the potential for relevant industry or regulatory documentation to exist outside the peer reviewed literature.

## Conclusions and Recommendations

Natural gas odorants have been critical for the detection of leaks that have saved lives and property while supporting the wide-spread availability of natural gas as a distributed source of energy. However, odorants that are intended to be transiently inhaled for leak detection and public safety may pose a risk to human health at exposures much lower than currently suspected. Overall, our review found limited evidence of both transient non-specific self-reported health symptoms and clinically diagnosed causative neurotoxic effects associated with odorant exposure. Additional weight of evidence is required to disentangle potential specific toxicological effects from transient malodor responses and/or generalized stress responses.

Few peer-reviewed toxicological and epidemiological studies have been conducted on commonly used odorants, especially at chronic, low-dose levels. The peer reviewed literature largely focuses on acute toxicity at high doses, and retrospective studies of lower-level odorant exposures in humans have been limited to accidental community exposure events and low sample-size case reports in occupational settings typically involving chemical spills. However, accidental environmental releases of odorants across documented community- and occupational exposure events are illustrative and do indicate a consistent symptom constellation ranging from mild to moderately debilitating including (in ranked order of prevalence) headache, eye irritation, nose and throat irritation, respiratory complaints such as shortness of breath and asthma attacks, and dermal irritation and rash. Of these, respiratory irritation and asthma exacerbations may be most debilitating whereas the high prevalence of eye irritation and skin rashes may indicate direct effects on exposed tissues in addition to exposure via inhalation.

Given its widespread use and intended purpose as an inhalant malodor, there is a surprising lack of established health and exposure benchmarks including but not limited to PELs, RELs, or TVLs. Recently, the TCEQ has developed interim short-term and long-term ESLs for the general public for commonly used odorants that generally ranged from 100-2,000 times lower than occupational limits where comparisons were available. Based on limited data on end-use natural gas odorant concentrations, detectable behind-the-meter leaks likely contain odorants at concentrations multiple orders of magnitude less than the long-term interim TCEQ ESLs. Furthermore, current federal regulations on combustible gas odorization state that, “The odorant may not be deleterious to persons…” [89] without further context related to ambient exposure limits. Although potential exposures may seem below the level of concern based upon these guidelines, current evidence indicates that organosulfur exposures can induce a range of debilitating symptoms at concentrations above human odor detection thresholds (e.g., <0.1ppb) and below ambient monitoring instrument detection limits (~5 ppb) suggesting that the short-term ESLs (4.9-500 ppb) proposed by the TCEQ would likely not protect against similar symptom onset.

Certain occupational settings may produce exposure regimes similar to or greater than the community exposure events reviewed here (e.g., among gas utility and commercial kitchen workers) [64••], yet few exposure- or health effect estimate studies have evaluated occupational settings. Additional research should also focus on the risks of odorant exposure among potentially susceptible populations, such as people with asthma, pregnant people, and children, as well as the potential health impacts of IPM given its frequent use and lack of research on its safety. This may require improvements in continuous monitoring equipment so that low concentrations relevant to chronic exposures can be measured.

Current hazardous material regulations provide guidance for the concentration of ethyl mercaptan acceptable for use as an odorant in LPG in the U.S., indicating that similar guidelines could exist for natural gas systems. Odorant concentration guidelines exist for other countries such as the the E.U. and United Kingdom, whereby “gas shall be odorized with 80% TBM and 20% DMS at an odorant injection rate of 6 mg/m^3^ which cannot vary by more than +- 2 mg/m^3^” [90]. To ensure the safety of individuals and populations exposed to natural gas leaks, we recommend that guidance on acceptable odorants and their concentrations be adopted following requisite study to determine odorant exposure levels that minimize symptom onset while maintaining malodor efficacy. Industry should also be required to publicly disclose the identities and concentrations of odorants added to natural gas and concentrations delivered to end users. We also recommend disclosure of odorant type and storage to first responders in the event of an environmental release. Emergency preparedness for the general public in the event of spills and leaks can also be improved, including ensuring that natural gas users are aware of the smell of natural gas and understand proper responses to natural gas leaks.

To improve understanding and management of natural gas odorants with respect to human health, we recommend the following: 1) require disclosure of odorants used and develop corresponding health-based concentration standards for odorizing natural gas beyond the lenient 1/5^th^ explosion risk end-point only; 2) studies are needed to better elucidate the distinction between malodors and any toxicological effects of odorant exposure including effects on potentially susceptible populations or subgroups with pre-existing respiratory conditions, or those that may exhibit some forms of odorant hypersensitivity or anosmia; 3) explicit risk assessments should be undertaken to develop occupational- and general exposure limits for commonly used odorants beyond the use of surrogate compounds; 4) improved real-time organosulfur or methane sensor detectors are needed with detection limits at very low concentrations with applications akin to smoke detectors or carbon monoxide detectors, particularly for high occupancy buildings, commercial kitchens, gas utility work sites, and settings where individual may suffer from a degree of anosmia; 5) increasing the concentration of odorants added to certain natural gas distribution systems could potentially improve leak detection of smaller natural gas leaks to help address methane leakage from a climate standpoint, though this could introduce additional occupational hazards or equity and environmental justice issues.

## Supplementary Information

ESM 1
