# Supplementary material for: Natural gas odorants: A scoping review of health effects
Source: Curr Environ Health Rep. 2023 Jul 25;10(3):337–52. doi: 10.1007/s40572-023-00403-w (PMC10504204; doi:10.1007/s40572-023-00403-w)
Supplement: Supplementary file 1 — ESM 1 [file 40572_2023_403_MOESM1_ESM.pdf]

SUPPLEMENTAL INFORMATION

Natural gas odorants: A scoping review of health effects

Current Environmental Health Reports

Drew R. Michanowicz*, Olivia M. Leventhal, Jeremy K. Domen, Samuel R. Williams, Eric D. Lebel, Lee Ann L. Hill, Jonathan J. Buonocore, Curtis L. Nordgaard, Aaron S. Bernstein, Seth B.C. Shonkoff

*Corresponding author

Corresponding author contact information:
Dr. Drew Michanowicz
PSE Healthy Energy
drew@psehealthyenergy.org
1440 Broadway, Suite 750
Oakland, California 94612

Methods - Scoping Review Search Terms and Screening Results

The following search terms were used to search the PubMed and Web of Science databases: (Mercaptan* OR "natural gas odorants" OR "tertiary butyl mercaptan" OR "tert-Butyl mercaptan" OR "2-Methyl-2-propanethiol" OR "2-Methylpropane-2-thiol" OR "tert-Butylthiol" OR "t-BuSH" OR "Isopropyl Mercaptan" OR "2-Propanethiol" OR "Propane-2-thiol" OR "Isopropanethiol" OR "2-propyl mercaptan" OR "Normal Propyl Mercaptan" OR "1-Propanethiol" OR "Propane-1-thiol" OR "Propyl mercaptan" OR "n-Propyl mercaptan" OR "Dimethyl Sulfide" OR "Tetrahydrothiophene") AND (expos* OR "inhalation exposure" OR inhal* OR "health" OR "side effects" OR symptom* OR toxic* OR "toxicological screening" OR fatal* OR coma* OR "death" OR pathogen* OR hazard* OR "risk" OR epidemiolog* OR "safety" OR carcin* OR "endocrine" OR dose*).

The results of the database search and article screening steps are shown in Figure S1. The list of all 22 articles that met inclusion criteria for full review are provided in Table S1.

Discussion - Olfactory dysfunction

Olfactory dysfunction due to aging, medical treatments, or medical conditions—such as the coronavirus disease 2019 (COVID-19)—may also be an important determinant of exposure to odorants due to an inability to detect or identify odors. An estimated 1.4% of Americans suffer from some form of impaired olfaction that worsens with age [1]. Notably, older populations have reduced ability to detect and identify natural gas odorants [2–9], with 31% of adults aged ≥70 years misidentifying natural gas odors in the 2012 United States National Health and Nutrition Examination Survey [9]. Human odorant detection thresholds also vary based on the odorant

compound itself, volume of inhalation, odorant dilution, experimental protocol, familiarity with surroundings, and mental distractions [2–4,10–12]. Misidentification of odorants may cause natural gas leaks to go unaddressed, increasing the risk of a leak-related explosion hazard. Although few studies have specifically examined the effects of COVID-19 associated loss of olfaction on natural gas detection, multiple studies cite the inability to detect natural gas leaks as a potential risk [13–16] and early study results indicate a decrease in ability to detect natural gas odorants in patients who have recovered from COVID-19 [17]. Other factors that may result in olfactory dysfunction include active chemotherapy, head trauma, and chronic sinusitis or persistent asthma with nasal polyps [18–22].

Large variances in human odor detection thresholds for many odorants (see Table 1), along with the odor recognition thresholds being 2-10 times higher than odor detection thresholds, also confound research of health outcomes related to exposure, along with setting appropriate odorant concentrations limits [23]. Furthermore, odor masking from competing odors or conditions such as olfactory adaptation (i.e., olfactory fatigue) may interfere with the ability to detect odorants. Olfactory adaptation occurs when continuous or repeated exposure to an odorant decreases olfactory sensitivity to the odorant [24]. Olfactory adaptation to organosulfur compounds has been demonstrated [12,25,26], and may occur with low-level chronic indoor leaks and occupational exposure, where a gradual build-up and continuous exposure to odorants is more likely. Given the wide range in odorant sensitivities in the general population, more research is needed related to odor detection thresholds for certain subgroups that may exhibit some forms of either anosmia or hypersensitivity.

Table S1. List of articles that met inclusion criteria for full review

Reference	Odorant(s)	Study type	Exposure Pathway	Location
Ljunggren and Norberg, 1943 [27] [a]	DMS, MM, DMDS	Animal - rats	Inhalation	N/A
Almeida et al. 2008 [28]	DMS	Animal - rats	Intraperitoneal	N/A
Zieve et al. 1974 [29]	DMS, MM, EM	Animal - rats	Inhalation	N/A
Butterworth et al. 1975 [30]	DMS	Animal - rats	Inhalation	N/A
Vahlkamp et al., 1979 [31]	DMS, MM, EM	Animal – rats	In-vitro	N/A
Guan et al., 2017 [32]	DMS	Animal – *C.elegans, Drosophila*	In-vivo	N/A
Georgieff and Turnovska, 1999 [33]	DMS, MM, H2S, etc.	Human - community exposure	Inhalation	Bulgaria
Jaakkola et al. 1990 [34]	DMS, etc.	Human - community exposure	Inhalation	Finland
Terazawa et al. 1991 [35]	DMS	Human - occupational; animal - rats	Inhalation	Japan
Kangas et al. 1984 [36]	DMS, H2S, MM	Human - occupational	Inhalation	Finland
Tansy et al. 1981 [37]	DMS, MM, DMDS	Animal - rats	Inhalation	N/A
Jeffery et al. 1988 [38]	DMS, dimethyl sulfoxide	Animal - mice & rats	In-vitro	N/A
Kocsis et al. 1975 [39]	DMS, dimethyl sulfoxide	Animal - rats	Intraperitoneal	N/A
Vernie et al. 1983 [40]	IPM	Cell culture	In-vitro	N/A
Ames and Stratton, 1991 [41]	NPM	Human - community exposure	Inhalation	Dorris, CA
U.S. Department of Health and Human Services, 2008 [42][b]	NPM	Human - community exposure	Inhalation	Fairburn, GA
Fairchild and Stokinger, 1958 [43]	NPM, TBM	Animal - rats & mice	Oral and intraperitoneal	N/A
Maciejewski et al. 2008 [44]	NPM	Animal - cats & dogs	Inhalation	Fairburn, GA

Los Angeles County Department of Public Health, 2016 [45][c]	TBM, THT	Human - community exposure	Inhalation	Aliso Canyon, CA
Behbod et al. 2014 [46]	TBM	Human - community exposure	Inhalation	Eight Mile, AL
Baur and Bittner, 2009 [47]	THT	Human - occupational	Inhalation and dermal	N/A
Chan et al. 2017 [48]	THT	Human - community exposure	Inhalation	Hong Kong

a. Included from citations in other papers
b. Study mentioned, but not cited in Maciejewski et al. 2008
c. Not included in literature search or citations

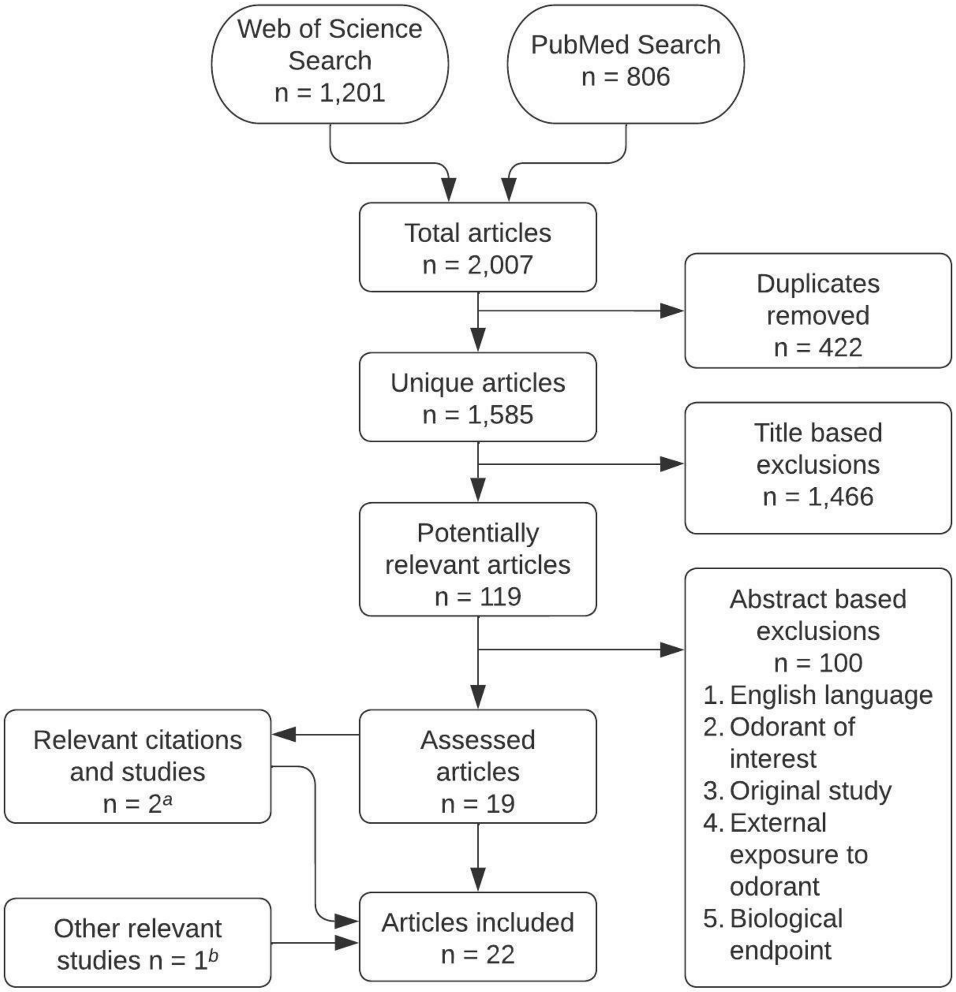

Figure S1. Flow chart of the review process with the number of articles included and rejected at each stage.

a. Does not include citations and studies that were inaccessible and could not be fully evaluated.

b. Government document not included in literature review related to Aliso Canyon, CA.

References

[1] Miwa T, Furukawa M, Tsukatani T, Costanzo RM, DiNardo LJ, Reiter ER. Impact of olfactory impairment on quality of life and disability. Archives of Otolaryngology–Head & Neck Surgery 2001;127:497–503. https://doi.org/10.1001/archotol.127.5.497.

[2] Stevens JC, Cain WS, Weinstein DE, Pierce JB. Aging impairs the ability to detect gas odor. Fire Technology 1987;23:198–204. https://doi.org/10.1007/BF01036936.

[3] Chalke H, Dewhurst J, Ward C, others. Loss of Sense of Smell in Old People. A Possible Contributory Factor in Accidental Poisoning from Town Gas. Public Health 1958;72:223–30. https://doi.org/10.1016/S0033-3506(58)80053-0.

[4] Jang SS, Choi JS, Kim JH, Kim N, Ference EH. Discordance Between Subjective and Objective Measures of Smell and Taste in US Adults. Otolaryngology–Head and Neck Surgery 2021:01945998211018386. https://doi.org/10.1177/019459982110183.

[5] Larsson M, Finkel D, Pedersen NL. Odor identification: influences of age, gender, cognition, and personality. The Journals of Gerontology Series B: Psychological Sciences and Social Sciences 2000;55:304–10. https://doi.org/10.1093/geronb/55.5.P304.

[6] Wysocki CJ, Gilbert AN. National Geographic Smell Survey: effects of age are heterogenous. Annals of the New York Academy of Sciences 1989;561:12–28. https://doi.org/10.1111/j.1749-6632.1989.tb20966.x.

[7] Cain W, Gent J, Commetto-Muniz J. The National Geographic Smell Survey and gas warning odor: Replication and extension. Chemical Senses 1993;18:536–7.

[8] Cain WS, Turk A. Smell of danger: an analysis of LP-gas odorization. American Industrial Hygiene Association Journal 1985;46:115–26. https://doi.org/10.1080/15298668591394527.

[9] Hoffman HJ, Rawal S, Li C-M, Duffy VB. New chemosensory component in the US National Health and Nutrition Examination Survey (NHANES): first-year results for measured olfactory dysfunction. Reviews in Endocrine and Metabolic Disorders 2016;17:221–40. https://doi.org/10.1007/s11154-016-9364-1.

[10] Abraham MH, Sánchez-Moreno R, Cometto-Muñiz JE, Cain WS. An algorithm for 353 odor detection thresholds in humans. Chemical Senses 2012;37:207–18. https://doi.org/10.1093/chemse/bjr094.

[11] Whisman ML, Goetzinger JW, Cotton FO, Brinkman DW. Odorant evaluation: A study of ethanethiol and tetrahydrothiophene as warning agents in propane. Environmental Science & Technology 1978;12:1285–8. https://doi.org/10.1021/es60147a003.

[12] Berglund B, Berglund U, Lindvall T. Olfactory self-and cross-adaptation: Effects of time of adaptation on perceived odor intensity. Sensory Processes 1978;2:191–7.

[13] Desai M, Oppenheimer J. The importance of considering olfactory dysfunction during the COVID-19 pandemic and in clinical practice. The Journal of Allergy and Clinical Immunology: In Practice 2020;9:7–12.

[14] Cameron EL, Møller P, Karn KS. Effects of COVID-19 on Sense of Smell: Human Factors/Ergonomics Considerations. Human Factors 2021:0018720821990162. https://doi.org/10.1177/0018720821990162.

[15] Soler ZM, Patel ZM, Turner JH, Holbrook EH. A primer on viral-associated olfactory loss in the era of COVID-19. International forum of allergy & rhinology, vol. 10, Wiley Online Library; 2020, p. 814–20. https://doi.org/10.1002/alr.22578.

[16] Khan AM, Kallogjeri D, Piccirillo JF. Growing Public Health Concern of COVID-19 Chronic Olfactory Dysfunction. JAMA Otolaryngology–Head & Neck Surgery 2021. https://doi.org/10.1001/jamaoto.2021.3379.

[17] Rebholz H, Pfaffeneder-Mantai F, Knoll W, Hassel A, Frank W, Kleber C. Olfactory dysfunction in SARS-CoV-2 infection: Focus on odorant specificity and chronic persistence. American Journal of Otolaryngology 2021;42:103014. https://doi.org/10.1016/j.amjoto.2021.103014.
[18] Liu G, Zong G, Doty RL, Sun Q. Prevalence and risk factors of taste and smell impairment in a nationwide representative sample of the US population: a cross-sectional study. Bmj Open 2016;6:e013246. http://dx.doi.org/10.1136/bmjopen-2016-013246.
[19] Deems DA, Doty RL, Settle RG, Moore-Gillon V, Shaman P, Mester AF, et al. Smell and taste disorders, a study of 750 patients from the University of Pennsylvania Smell and Taste Center. Archives of Otolaryngology–Head & Neck Surgery 1991;117:519–28. https://doi.org/10.1001/archotol.1991.01870170065015.
[20] Langdon C, Alobid I, Quinto L, Valero A, Picado C, Marin C, et al. Self-perception of olfactory dysfunction is associated with history of Traumatic Brain Injury: post-hoc analysis from the OLFACAT survey. Rhinology 2019;57:460–8. https://doi.org/10.4193/rhin18.295.
[21] Alobid I, Bernal-Sprekelsen M, Mullol J. Chronic rhinosinusitis and nasal polyps: the role of generic and specific questionnaires on assessing its impact on patient's quality of life. Allergy 2008;63:1267–79. https://doi.org/10.1111/j.1398-9995.2008.01828.x.
[22] Alobid I, Cardelus S, Benítez P, Guilemany JM, Roca-Ferrer J, Picado C, et al. Persistent asthma has an accumulative impact on the loss of smell in patients with nasal polyposis. Rhinology 2011;49:519–24.
[23] Hellman TM, Small FH. Characterization of the odor properties of 101 petrochemicals using sensory methods. Journal of the Air Pollution Control Association 1974;24:979–82. https://doi.org/10.1080/00022470.1974.10470005.
[24] Dalton P. Psychophysical and behavioral characteristics of olfactory adaptation. Chemical Senses 2000;25:487–92. https://doi.org/10.1093/chemse/25.4.487.
[25] Matsubasa T, Gotow N, Gomi Y, Kobayakawa T. A method for psychophysical screening of odorants for use in city gas based on olfactory adaptation tolerance. Chemosensory Perception 2016;9:120–30. https://doi.org/10.1007/s12078-016-9213-3.
[26] Moschandreas D, Jones D, Martinus J, Ragus E. Odor recognition and fatigue. IIT Research Inst., Chicago, IL (USA); 1982.
[27] Ljunggren G, Norberg B. On the Effect and Toxicity of Dimethyl Sulfide, Dimethyl Disulfide and Methyl Mercaptan. Acta Physiologica Scandinavica 1943;5:248–55. https://doi.org/10.1111/j.1748-1716.1943.tb02053.x.
[28] Almeida AF, Nation PN, Guidotti TL. Mechanism and Treatment of Sulfide-Induced Coma: A Rat Model. Int J Toxicol 2008;27:287–93. https://doi.org/10.1080/10915810802210166.
[29] Zieve L, Doizaki WM, Zieve J. Synergism between mercaptans and ammonia or fatty acids in the production of coma: a possible role for mercaptans in the pathogenesis of hepatic coma. J Lab Clin Med 1974;83:16–28.
[30] Butterworth KR, Carpanini FMB, Gaunt IF, Hardy J, Kiss IS, Gangolli SD. Short-term toxicity of dimethyl sulphide in the rat. Food and Cosmetics Toxicology 1975;13:15–22. https://doi.org/10.1016/0015-6264(75)90078-4.
[31] Vahlkamp T, Meijer A, Wilms J, Chamuleau R. Inhibition of mitochondrial electron transfer in rats by ethanethiol and methanethiol. Clinical Science (London, England: 1979) 1979;56:147–56.
[32] Guan X-L, Wu P-F, Wang S, Zhang J-J, Shen Z-C, Luo H, et al. Dimethyl sulfide protects against oxidative stress and extends lifespan via a methionine sulfoxide reductase A-

dependent catalytic mechanism. Aging Cell 2017;16:226–36. https://doi.org/10.1111/acel.12546.
[33] Georgieff D, Turnovska T. Influence of odour immissions from cellulose-paper industry on some health indicators. Folia Med (Plovdiv) 1999;41:38–42.
[34] Jaakkola JJ, Vilkka V, Marttila O, Jäppinen P, Haahtela T. The South Karelia Air Pollution Study. The effects of malodorous sulfur compounds from pulp mills on respiratory and other symptoms. Am Rev Respir Dis 1990;142:1344–50. https://doi.org/10.1164/ajrccm/142.6_Pt_1.1344.
[35] Terazawa K, Mizukami K, Wu B, Takatori T. Fatality due to inhalation of dimethyl sulfide in a confined space: a case report and animal experiments. Int J Legal Med 1991;104:141–4. https://doi.org/10.1007/BF01369718.
[36] Kangas J, Jäppinen P, Savolainen H. Exposure to hydrogen sulfide, mercaptans and sulfur dioxide in pulp industry. Am Ind Hyg Assoc J 1984;45:787–90. https://doi.org/10.1080/15298668491400647.
[37] Tansy MF, Kendall FM, Fantasia J, Landin WE, Oberly R, Sherman W. Acute and subchronic toxicity studies of rats exposed to vapors of methyl mercaptan and other reduced-sulfur compounds. J Toxicol Environ Health 1981;8:71–88. https://doi.org/10.1080/15287398109530051.
[38] Jeffery EH, Arndt K, Haschek WM. Mechanism of inhibition of hepatic bioactivation of paracetamol by dimethyl sulfoxide. Drug Metabol Drug Interact 1988;6:413–24. https://doi.org/10.1515/dmdi.1988.6.3-4.413.
[39] Kocsis JJ, Harkaway S, Snyder R. Biological effects of the metabolites of dimethyl sulfoxide. Ann N Y Acad Sci 1975;243:104–9. https://doi.org/10.1111/j.1749-6632.1975.tb25349.x.
[40] Vernie LN, De Vries M, Karreman L, Topp RJ, Bont WS. Inhibition of amino acid incorporation in a cell-free system and inhibition of protein synthesis in cultured cells by reaction products of selenite and thiols. Biochim Biophys Acta 1983;739:1–7. https://doi.org/10.1016/0167-4781(83)90037-4.
[41] Ames RG, Stratton JW. Acute health effects from community exposure to N-propyl mercaptan from an ethoprop (Mocap)-treated potato field in Siskiyou County, California. Arch Environ Health 1991;46:213–7. https://doi.org/10.1080/00039896.1991.9937451.
[42] U.S. Department of Health and Human Services. Health Consultation PSC Recovery Systems, Fairburn, Fulton County, Georgia 2008. https://www.atsdr.cdc.gov/HAC/pha/PSC_Recovery_Systems/PSCRecoverySystems030708.pdf (accessed October 14, 2021).
[43] Fairchild EJ, Stokinger HE. Toxicologic studies on organic sulfur compounds. I. Acute toxicity of some aliphatic and aromatic thiols (mercaptans). Am Ind Hyg Assoc J 1958;19:171–89. https://doi.org/10.1080/00028895809343573.
[44] Maciejewski R, Glickman N, Moore G, Zheng C, Tyner B, Cleveland W, et al. Companion animals as sentinels for community exposure to industrial chemicals: the Fairburn, GA, propyl mercaptan case study. Public Health Rep 2008;123:333–42. https://doi.org/10.1177/003335490812300313.
[45] Los Angeles County Department of Public Health. Aliso Canyon Gas Leak Community Assessment for Public Health Emergency Response (CASPER) 2016. http://publichealth.lacounty.gov/media/docs/CASPERFinalReport.pdf (accessed January 21, 2022).

[46] Behbod B, Parker EM, Jones EA, Bayleyegn T, Guarisco J, Morrison M, et al. Community health assessment following mercaptan spill: Eight Mile, Mobile County, Alabama, September 2012. J Public Health Manag Pract 2014;20:632–9. https://doi.org/10.1097/PHH.0000000000000024.
[47] Baur X, Bittner C. Occupational obstructive airway diseases caused by the natural gas odorant tetrahydrothiophene--two case reports. Am J Ind Med 2009;52:982–6. https://doi.org/10.1002/ajim.20761.
[48] Chan YC, Chan CK, Ng CH, Ng SH, Lau KK, Tse ML. Hong Kong Poison Information Centre: Annual report 2016. Hong Kong Journal of Emergency Medicine 2017;24:244–54. https://doi.org/10.1177/1024907917724.
